# A high-fidelity residential building occupancy detection dataset

**DOI:** 10.1038/s41597-021-01055-x

**Published:** 2021-10-28

**Authors:** Margarite Jacoby, Sin Yong Tan, Gregor Henze, Soumik Sarkar

**Affiliations:** 1 University of Colorado Boulder, Department of Civil, Environmental and Architectural Engineering, Boulder, 80309-0428 United States; 2 Iowa State University, Department of Mechanical Engineering, Ames, 50011 United States; 3 National Renewable Energy Laboratory, Golden, 80401 United States; 4 Renewable and Sustainable Energy Institute, Boulder, 80309 United States

**Keywords:** Databases, Mechanical engineering, Energy supply and demand, Energy efficiency, Energy conservation

## Abstract

This paper describes development of a data acquisition system used to capture a range of occupancy related modalities from single-family residences, along with the dataset that was generated. The publicly available dataset includes: grayscale images at 32-by-32 pixels, captured every second; audio files, which have undergone processing to remove personally identifiable information; indoor environmental readings, captured every ten seconds; and ground truth binary occupancy status. The data acquisition system, coined the mobile human presence detection (HPDmobile) system, was deployed in six homes for a minimum duration of one month each, and captured all modalities from at least four different locations concurrently inside each home. The environmental modalities are available as captured, but to preserve the privacy and identity of the occupants, images were downsized and audio files went through a series of processing steps, as described in this paper. This dataset adds to a very small body of existing data, with applications to energy efficiency and indoor environmental quality.

## Abstract (summary)

**Table:** 

Measurement(s)	room temperature ambient air • room air relative humidity • Carbon Dioxide • total volatile organic compounds • room illuminance • Audio Media • Digital Photography • Occupancy
Technology Type(s)	Thermostat Device • humidity sensor • gas sensor • light sensor • Microphone Device • Camera Device • manual recording
Sample Characteristic - Environment	residential building
Sample Characteristic - Location	United States of America

Machine-accessible metadata file describing the reported data: 10.6084/m9.figshare.14920131

## Background & Summary

Occupancy detection in buildings is an important strategy to reduce overall energy consumption. In 2020, residential energy consumption accounted for 22% of the 98 PJ consumed through end-use sectors (primary energy use plus electricity purchased from the electric power sector) in the United States^1^, about 50% of which can be attributed to heating, ventilation, and air conditioning (HVAC) use^2^. Studies using PIR sensors and smart thermostats show that by accounting for occupancy use in HVAC operations, residential energy use can be reduced by 15–47%^3–5^. Other studies show that by including occupancy information in model predictive control strategies, residential energy use could be reduced by 13–39%^6,7^. While these reductions are not feasible in all climates, as humidity or freezing risk could make running HVAC equipment a necessity during unoccupied times, moderate temperature setbacks as a result of vacancy information could still lead to some energy savings. Additional benefits of occupancy detection in homes include enhanced occupant comfort, home security, and home health applications^8^.

Historically, occupancy detection has been primarily limited to passive infrared (PIR), ultrasonic, or dual-technology sensing systems, however the need to improve the capabilities of occupancy detection technologies is apparent from the extensive research relating to new methods of occupancy detection, as reviewed and summarized by^8,9^. Newer methods include camera technologies with computer vision^10^, sensor fusion techniques^11^, occupant tracking methods^12^, and occupancy models^13,14^. Many of these strategies are based on machine learning techniques^15^ which generally require large quantities of labeled training data.

Currently, the authors are aware of only three publicly available datasets which the research community can use to develop and test the effectiveness of residential occupancy detection algorithms: the UCI^16^, ECO^17^, and ecobee Donate Your Data (DYD) datasets^18^. The UCI dataset captures temperature, relative humidity, light levels, and CO_2_ as features recorded at one minute intervals. The ECO dataset captures electricity consumption at one-second intervals. The DYD data is collected from ecobee thermostats, and includes environmental and system measurements such as: runtime of heating and cooling sources, indoor and outdoor relative humidity and temperature readings, detected motion, and thermostat schedules and setpoints. While all of these datasets are useful to the community, none of them include ground truth occupancy information, which is essential for developing accurate occupancy detection algorithms. Additionally, other indoor sensing modalities, which these datasets do not capture, are also desirable. For example, images and audio can both provide strong indications of human presence. While many datasets exist for the use of object (person) detection, person recognition, and people counting in *commercial* spaces^19–21^, the authors are aware of no publicly available datasets which capture these modalities for *residential* spaces. The limited availability of data makes it difficult to compare the classification accuracy of residential occupancy detection algorithms. It is understandable, however, why no datasets containing images and audio exist, as privacy concerns make capturing and publishing these data types difficult^22^. Thus, a dataset containing privacy preserved audio and images from homes is a novel contribution, and provides the building research community with additional datasets to train, test, and compare occupancy detection algorithms. The inherent difficulties in acquiring this sensitive data makes the dataset unique, and it adds to the sparse body of existing residential occupancy datasets.

The data described in this paper was collected for use in a research project funded by the Advanced Research Projects Agency - Energy (ARPA-E). The project was part of the Saving Energy Nationwide in Structures with Occupancy Recognition (SENSOR) program, which was launched in 2017 to “develop user-transparent sensor systems that accurately quantify human presence to dramatically reduce energy use in commercial and residential buildings”^23^. Our team is specifically focused on residential buildings and we are using the captured data to inform the development of machine learning algorithms along with novel RFID-based wireless and battery-free hardware for occupancy detection. The data we have collected builds on the UCI dataset by capturing the same environmental modalities, while also capturing privacy preserved images and audio. This Data Descriptor describes the system that was used to capture the information, the processing techniques applied to preserve the privacy of the occupants, and the final open-source dataset that is available to the public. See Table 1 for a summary of modalities captured and available.

**Table 1:** Summary of all modalities as collected by the data acquisition system and as available for download.

Measurement	Units or Description	Accuracy	Precision	Range
Indoor Air Temperature	Degree Celsius (°C)	±0.2 °C	±0.1 °C	−40–125 °C
Relative Humidity	Percentage (% rH)	±0.2%rH	±0.1%rH	0–100%
Carbon Dioxide Equivalence	Parts per million (ppm)	≈±15%^†^	±1−31 ppm	400–60,000 ppm
Total Volatile Organic Compounds	Parts per billion (ppb)	≈±10%^†^	±1−32 ppb	0–60,000 ppb
Ambient light levels	Lux (lx)	≈±45%^†^	unspecified	unspecified
Audio (as collected)	10-second WAV file	N/A	18 bit	N/A
Images (as collected)	112k pixel b&w PNG file	N/A	36 dB S/N ratio	N/A
Audio (as available)	Audio signal as CSV file	N/A	N/A	N/A
Images (as available)	1,024 pixel b&w PNG file	N/A	N/A	N/A

Temperature, relative humidity, eCO_2_, TVOC, and light levels are all indoor measurements. Accuracy, precision, and range are as specified by the sensor product sheets. ^†^indicates that the true value is within the specified percentage of the measured value, as outlined in the product sheets.

## Methods

Time series data related to occupancy were captured over the course of one-year from six different residences in Boulder, Colorado. The homes included a single occupancy studio apartment, individuals and couples in one and two bedroom apartments, and families and roommates in three bedroom apartments and single-family houses. The homes and apartments tested were all of standard construction, representative of the area’s building stock, and were constructed between the 1960’s and early 2000’s. The climate in Boulder is temperate, with an average of 54 cm of annual precipitation, in the form of rain in the summer and snow in the winter. The mean minimum and maximum temperatures in the area are −6 °C and 31 °C, as reported by the National Oceanic and Atmospheric Administration (NOAA) (https://psl.noaa.gov/boulder).

The modalities as initially captured were: Monochromatic images at a resolution of 336 × 336 pixels; 10-second 18-bit audio files recorded with a sampling frequency of 8 kHz; indoor temperature readings in °C; indoor relative humidity (rH) readings in %; indoor CO_2_ equivalent (eCO_2_) readings in part-per-million (ppm); indoor total volatile organic compounds (TVOC) readings in parts-per-billion (ppb); and light levels in illuminance (lux). Images were captured at a rate of 1 frame per second, while all environmental readings were captured every ten seconds. The ten-second sampling frequency of the environmental sensors was greater than would be necessary to capture dynamics such as temperature changes, however this high frequency was chosen to allow researchers the flexibility of choosing their own down-sampling methods, and to potentially capture occupancy related events such as lights being turned on. Audio files were captured back to back, resulting in 8,640 audio files per day. Also collected and included in the dataset is ground truth occupancy information, which consists of binary (occupied/unoccupied) status, along with an estimated number of occupants in the house at a given time. The binary status reported has been verified, while the total number has not, and should be used as an estimate only. In addition, zone-labels are provided for images, which indicate with a binary flag whether each image shows a person or not. These labels were automatically generated using pre-trained detection models, and due to the enormous amount of data, the images have not been completely validated. Instead, they have been spot-checked and metrics for the accuracy of these labels are provided.

Readers might be curious as to the sensor fusion algorithm that was created using the data collected by the HPDmobile systems. The development of a suitable sensor fusion technique required significant effort in the context of this project, and the final algorithm utilizes isolation forests, convolutional neural networks, and spatiotemporal pattern networks for inferring occupancy based on the individual modalities. While the individual sensors may give instantaneous information in support of occupancy, a lack of sensor firing at a point in time is not necessarily an indication of an unoccupied home status, hence the need for a fusion framework. Our best fusion algorithm is one which considers both concurrent sensor readings, as well as time-lagged occupancy predictions. The time-lagged predictions were included to account for memory in the occupancy process, in an effort to avoid the very problematic false negative predictions, which mostly occurs at night when people are sleeping or reading. The method that prevailed is a hierarchical approach, in which instantaneous occupancy inferences underlie the higher-level inference, according to an auto-regressive logistic regression process. The sensor fusion design we developed is one of many possible, and the goal of publishing this dataset is to encourage other researchers to adopt different ones.

### Data acquisition system

Fundamental to the project was the capture of (1) audio signals with the capacity to recognize human speech (ranging from 100 Hz to 4 kHz) and (2) monochromatic images of at least 10,000 pixels. Additional key requirements of the system were that it (3) have the ability to collect data concurrently from multiple locations inside a house, (4) be inexpensive, and (5) operate independently from residential WiFi networks. Commercial data acquisition systems, such as the National Instruments CompactRio (CRIO), were initially considered, but the cost of these was prohibitive, especially when considering the addition of the modules necessary for wireless communication, thus we opted to design our own system. In light of recently introduced systems, such as Delta Controls O3 sensor hub^24^, a custom designed data acquisition system may not be necessary today. Two independent systems were built so data could be captured from two homes simultaneously. The final systems, each termed a Mobile Human Presence Detection system, or *HPDmobile*, are built upon Raspberry Pi single-board computers (referred to as SBCs for the remainder of this paper), which act as sensor hubs, and utilize inexpensive sensors and components marketed for hobby electronics.

Each HPDmobile data acquisition system consists of:

- Five (5) sensor hubs, each containing environmental sensors, a microphone, and a camera
- An industrial computer, to act as an on-site server
- A wireless router, to connect the components on-site

**Fig. 1:**
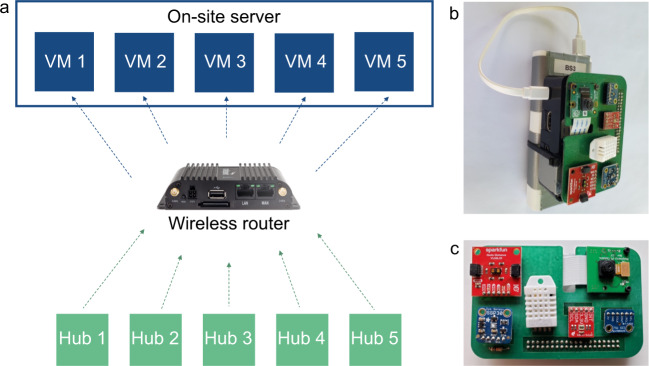
(**a**) System architecture, hardware components, and network connections of the HPDmobile data acquisition system. (**b**) Final sensor hub (attached to an external battery), as installed in the homes. (**c**) Custom designed printed circuit board with sensors attached. Sensors, clockwise from top right, are: camera, microphone, light, temperature/humidity, gas (CO_2_ and TVOC), and distance.

The sensor hubs run a Linux based operating system and serve to collect and temporarily store individual sensor readings. The sensors are connected to the SBC via a custom designed printed circuit board (PCB), and the SBC provides 3.3 Vdc power to all sensors. Each sensor hub is connected to an on-site server through a wireless router, all of which are located inside the home being monitored. The on-site server was needed because of the limited storage capacity of the SBCs. The server runs a separate Linux-based virtual machine (VM) for each sensor hub. Data that are captured on the sensor hub are periodically transmitted wirelessly to the accompanying VM, where they are stored for the duration of the testing period in that home. See Fig. 1a for a diagram of the hardware and network connections. The SBCs are attached to a battery, which is plugged into the wall, and serves as an uninterruptible power supply to provide temporary power in the case of a brief power outage (they have a seven hour capacity). The batteries also help enable the set-up of the system, as placement of sensor hubs can be determined by monitoring the camera output before power-cords are connected. See Fig. 1b,c for images of the full sensor hub and the completed board with sensors. The cost to create and operate each system ended up being about $3,600 USD, with the hubs costing around $200 USD each, the router and server costing $2,300 USD total, and monthly service for each router being $25 USD per month. Research, design, and testing of the system took place over a period of six months, and data collection with both systems took place over one year.

### Ground truth establishment

To ensure accuracy, ground truth occupancy was collected in two manners. First, a geo-fence was deployed for all test homes. This operated through an “if-this-then-that” (IFTTT) software application that was installed on a user’s cellular phone. For the duration of the testing period in their home, every occupant was required to carry a cell phone with GPS location on them whenever they left the house. When they entered or exited the perimeter of the home, the IFTTT application triggered and registered the event type (exit or enter), the user, and the timestamp of the occurrence. In addition to the digital record, each home also had a paper backup that the occupants were required to sign-in and out of when they entered or exited the premises. At the end of the collection period, occupancy logs from the two methods (paper and digital) were reviewed, and any discrepancies or questionable entries were verified or reconciled with the occupants. Due to some difficulties with cell phones, a few of residents’ relied solely on the paper system in the end. The occupancy logs for all residents and guests were combined in order to generate a binary occupied/unoccupied status for the whole-house.

### Field implementation

Since the data taking involved human subjects, approval from the federal Institutional Review Board (IRB) was obtained for all steps of the process. This meant that a Human Subject Research (HSR) plan was in place before any data taking began, and ensured that strict protocols were followed regarding both collection of the data and usage of it. Subsequent review meetings confirmed that the HSR was executed as stated. Additional IRB approval was sought and granted for public release of the dataset after the processing methods were finalized. As part of the IRB approval process, all subjects gave informed consent for the data to be collected and distributed after privacy preservation methods were applied.

Test subjects were recruited from the testing university’s department of architectural engineering graduate students and faculty in the front range of Colorado. Test homes were chosen to represent a variety of living arrangements and occupancy styles. The homes tested consisted of stand-alone single family homes and apartments in both large and small complexes. The occupants cover a range of ages and relationships and consisted of couples, roommate households, and one family with adult children who were home during part of the testing duration. Because of IRB restrictions, no homes with children under the age of 18 were included. Three of the six homes had pets - both indoor and outdoor cats and one dog. See Table 2 for a summary of homes selected.

**Table 2:** Home and occupant attributes.

ID	Occupants	Pets	Type	Home Size (m^2^)	Area Monitored (%)	Hubs Deployed	Days	% Occ
H1	4 adults	1 cat	House	149	32	5	30	0.82
H2	1 adult	None	Apartment	72	53	4	17	0.61
H3	4 adults	1 dog	House	285	28	5	35	0.78
H4	3 adults	None	Apartment	93	36	5	17	0.82
H5	2 adults	1 cat	Apartment	54	48	5	15	0.76
H6	1 adult	None	Apartment	62	58	4	38	0.47

**Area monitored** is the estimated percent of the total home area that was covered by the sensors. **Days** refers to the number of days of data that were released from the home, while **% Occ** refers to the percentage of time the home was occupied by at least one person (for the days released).

**Fig. 2:**
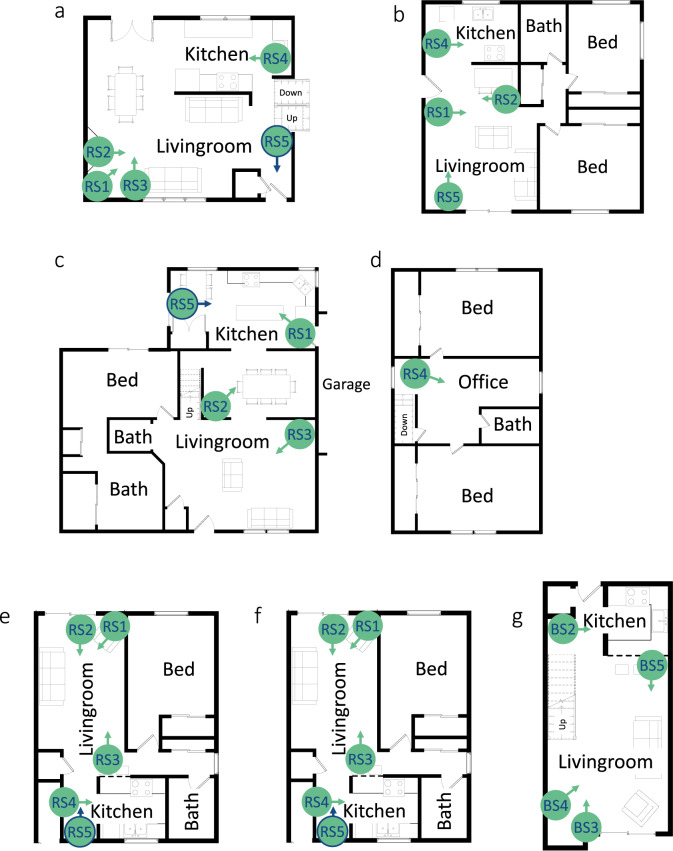
Home layouts and sensor placements. Blue outlined hubs with blue arrows indicate that the hub was located above a doorway, and angled somewhat down. (**a**) H1: Main level of three-level home. (**b**) H2: Full apartment layout. (**c**) and (**d**) H3: Main and top level (respectively) of three-level home. (**e**) H4: Main level of two-level apartment. (**f**) H5: Full apartment layout. (**g**) H6: Main level of studio apartment with lofted bedroom.

The number of sensor hubs deployed in a home varied from four to six, depending on the size of the living space. Hubs were placed only in the common areas, such as the living room and kitchen. In consideration of occupant privacy, hubs were not placed in or near bathrooms or bedrooms. Ideal hub locations were identified through conversations with the occupants about typical use patterns of the home. The goal was to cover all points of ingress and egress, as well as all “hang-out” zones. Hubs were placed either next to or facing front doors and in living rooms, dining rooms, family rooms, and kitchens. Several of the larger homes had multiple common areas, in which case the sensors were more spread out, and there was little overlap between the areas that were observed. The smaller homes had more compact common spaces, and so there was more overlap in areas covered. See Fig. 2 for home layouts with sensor hub locations marked.

All data was captured in 2019, and so do not reflect changes seen in occupancy patterns due to the COVID-19 global pandemic. Despite the relative normalcy of the data collection periods, occupancy in the homes is rather high (ranging from 47% to 82% total time occupied). This is most likely due to the relative homogeneity of the test subjects, and the fact that many were graduate students with atypical schedules, at least one of whom worked from home exclusively. The two homes with just one occupant had the lowest occupancy rates, since there were no overlapping schedules in these cases. The homes with pets had high occupancy rates, which could be due to pet owners needing to be home more often, but is likely just a coincidence. We also cannot discount the fact that occupants’ behavior might have been altered somewhat by the knowledge of monitoring, however, it seems unlikely that this knowledge would have led to increased occupancy rates.

### Data processing

After collection, data were processed in a number of ways. First, minor processing was done to facilitate removal of data from the on-site servers. Next, processing to validate the data and check for completeness was performed. Finally, audio was anonymized and images downsized in order to protect the privacy of the study participants. Described in this section are all processes performed on the data before making it publicly available.

#### Images

**Fig. 3:**
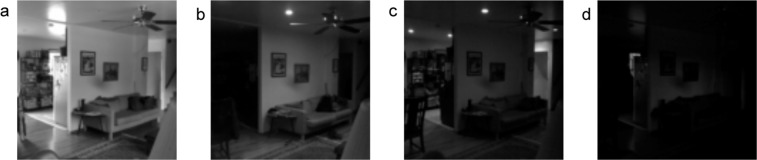
Four different images from the same sensor hub, comparing the relative “brightness” of the images, as described by the average pixel value. The images shown are 112 × 112 pixels. (**a**) Average pixel brightness: 106. (**b**) Average pixel brightness: 43. (**c**) Average pixel brightness: 32. (**d**) Average pixel brightness: 10.

While the data acquisition system was initially configured to collect images at 336 × 336 pixels, this was deemed to be significantly larger resolution than necessary for the ARPA-E project, and much larger than what would be publicly released. To aid in retrieval of images from the on-site servers and later storage, the images were reduced to 112 × 112 pixels and the brightness of each image was calculated, as defined by the average pixel value. Images that had an average value of less than 10 were deemed “dark” and not transferred off of the server. Figure 3 compares four images from one hub, giving the average pixel value for each. Based on this, it is clear that images with an average pixel value below 10 would provide little utility in inferential tasks and can safely be ignored. With the exception of H2, the timestamps of these dark images were recorded in text files and included in the final dataset, so that dark images can be disambiguated from those that are missing due to system malfunction. Since the hubs were collecting images 24-hours a day, dark images accounted for a significant portion of the total collected, and omitting these significantly reduces the size of the dataset. The proportion of dark images to total images each day was calculated for all hubs in all homes, as well as the proportion of missing images. Summaries of these can be found in Table 3.

**Table 3:** Summary of the completeness of data collected in each home.

Home	Hub	Audio	Env	Images	% Dark
H1	RS1	0.94	0.90	1.00	0.45
	RS2	0.97	0.93	1.00	0.01
	RS3	0.95	0.91	0.99	0.44
	RS4	0.96	0.92	1.00	0.48
	RS5	0.96	0.92	0.96	0.51
H2	RS1	0.90	0.93	1.00	0.30
	RS2	0.89	0.91	0.98	0.22
	RS4	0.92	0.92	1.00	0.00
	RS5	0.92	0.92	1.00	0.23
H3	RS1	0.95	0.90	1.00	0.37
	RS2	0.95	0.89	1.00	0.33
	RS3	0.97	0.92	1.00	0.33
	RS4	0.97	0.93	1.00	0.41
	RS5	0.97	0.91	1.00	0.38
H4	RS1	0.84	0.93	1.00	0.34
	RS2	0.95	0.94	0.97	0.35
	RS3	0.96	0.94	1.00	0.36
	RS4	0.86	0.91	0.89	0.34
	RS5	0.97	0.93	1.00	0.36
H5	RS1	0.22	0.21	1.00	0.32
	RS2	0.97	0.92	1.00	0.32
	RS3	0.96	0.92	0.99	0.31
	RS4	0.86	0.89	0.95	0.33
	RS5	0.89	0.91	1.00	0.33
H6	BS2	0.00	0.90	0.99	0.45
	BS3	0.92	0.92	0.95	0.38
	BS4	0.93	0.91	0.95	0.38
	BS5	0.93	0.90	0.96	0.37

Values given are the number of files collected for that modality in that location, relative to the total number that could be collected in a day, averaged over all the days that are presented in the final dataset. **Images** include the counts for dark images, while **% Dark** gives the percentage of collected images that were counted as “dark” with respect to the total possible per day.

**Fig. 4:**
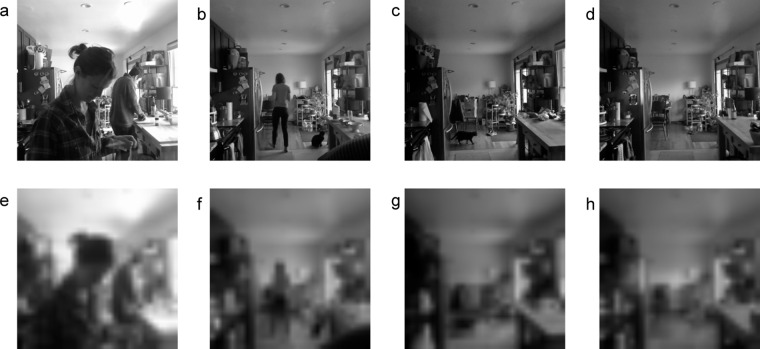
(**a**–**d**) Original captured images at 336 × 336 pixels. (**e**–**h**) Same images, downsized to 32 × 32 pixels. Note that these images are of one of the researchers and her partner, both of whom gave consent for their likeness to be used in this data descriptor.

As necessary to preserve the privacy of the residents and remove personally identifiable information (PII), the images were further downsized, from 112 × 112 pixels to 32 × 32 pixels, using a bilinear interpolation process. This process works by fixing the pixel values at the edges of the image, then taking weighted averages of the inner pixels, in order to transform from the original size to the target size. Thus new pixel values are generated from linear combinations of the original values. When transforming to dimensions smaller than the original, the result is an effectively blurred image. Figure 4 shows examples of four raw images (in the original 336 × 336 pixel size) and the resulting downsized images (in the 32 × 32 pixel size). Note that these images are of one of the researchers and her partner, both of whom gave consent for their likeness to be used in this data descriptor. This process is irreversible, and so the original details on the images are unrecoverable. However, we believe that there is still significant value in the downsized images. To increase the utility of the images, zone-based labels are provided for the images. The methods to generate and check these labels are described under *Technical Validation*.

#### Audio

**Fig. 5:**
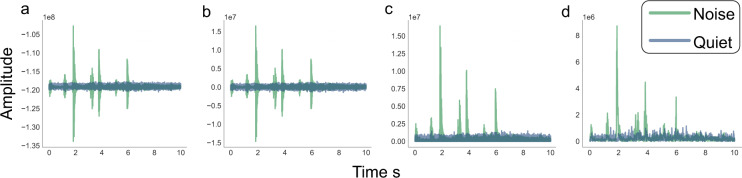
Audio processing steps performed on two audio files. In “noise” there is recognizable movement of a person in the space, while in “quiet” there are no audible sounds. (**a**) Raw waveform sampled at 8 kHz. (**b**) Waveform after applying a mean shift. (**c**) Waveform after full wave rectification. (**d**) Waveform after downsampling by integer factor of 100.

Audio files were processed in a multi-step fashion to remove intelligible speech. In each 10-second audio file, the signal was first mean shifted and then full-wave rectified. Finally, the signal was downsampled by a factor of 100 and the resulting audio signal was stored as a CSV file. See Fig. 5 for a visual of the audio processing steps performed. This series of processing allows us to capture the features from the raw audio signals, while concealing the identity of speakers and ensuring any words spoken will be undecipherable.

#### Environmental sensors

Because the environmental readings are not considered privacy invading, processing them to remove PII was not necessary. Minimal processing on the environmental data was performed only to consolidate the readings, which were initially captured in minute-wise JSON files, and to establish a uniform sampling rate, as occasional errors in the data writing process caused timestamps to not always fall at exact 10-second increments. Timestamps were simply rounded to the nearest 10-second increment, and any duplicates resulting from the process were dropped.

## Data Records

**Fig. 6:**
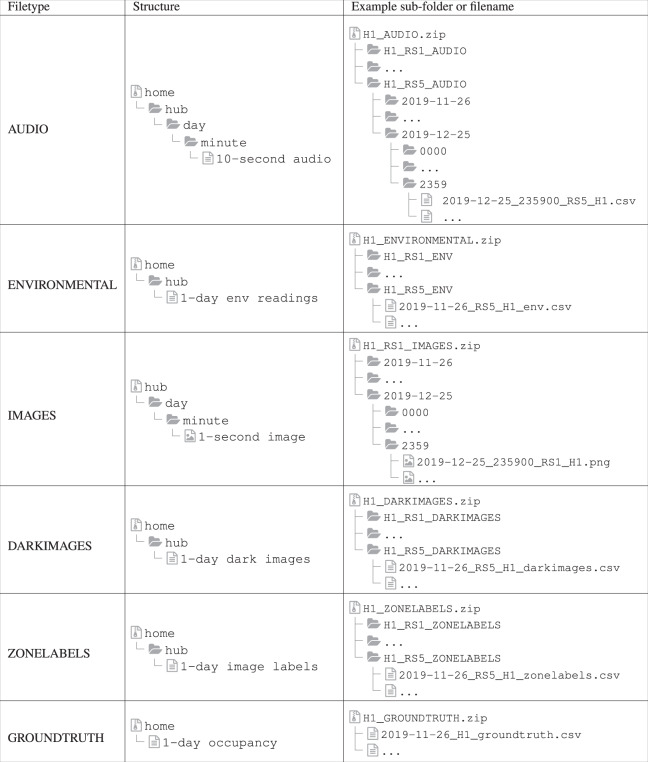
Example of the data records available for one home. The **Filetype** shows the top-level compressed files associated with this modality, while **Example sub-folder or filename** highlights one possible route to a base-level data record within that folder. **Structure** gives the tree structure of sub-directories, with the final entry in each section describing the data record type.

The released dataset is hosted on figshare^25^. Data for each home consists of audio, images, environmental modalities, and ground truth occupancy information, as well as lists of the dark images not included in the dataset. Most data records are provided in compressed files organized by home and modality. Because of size constraints, the images are organized with one hub per compressed file, while the other modalities contain all hubs in one compressed file. Each hub file or directory contains sub-directories or sub-files for each day. See Fig. 6 for a diagram of the folder structure with example folders and files.

Audio and image files are stored in further sub-folders organized by minute, with a maximum of 1,440 minute folders in each day directory. Each audio minute folder contains a maximum of six CSV files, each representing a processed ten-second audio clip from one hub, while each image minute folder contains a maximum of 60 images in PNG format. Lists of dark images are stored in CSV files, organized by hub and by day. Each day-wise CSV file contains a list of all timestamps in the day that had an average brightness of less than 10, and was thus not included in the final dataset. Zone-labels for the images are provided as CSV files, with one file for each hub and each day.

Environmental data are stored in CSV files, with one day’s readings from a single hub in each CSV. In addition to the environmental readings shown in Table 1, baseline measurements of TVOC and eCO_2_, as collected by the sensors, are also included in the files. Ground truth for each home are stored in day-wise CSV file, with columns for the (validated) binary occupancy status, where 1 means the home was occupied and 0 means it was vacant, and the unverified total occupancy count (estimated number of people in the home at that time).

In the process of consolidating the environmental readings, placeholder timestamps were generated for missing readings, and so each day-wise CSV contains exactly 8,640 rows of data (plus a header row), although some of the entries are empty. Missing data are represented as blank, unfilled cells in the CSVs. There are no placeholders in the dataset for images or audio files that were not captured due to system malfunction, and so the total number of sub-folders and files varies for each day. See Table 3 for the average number of files captured by each hub.

Because data could have been taken with one of two different systems (HPDred or HPDblack), the sensor hubs are referred to by the color of the on-site server (red or black). E.g., the first hub in the red system is called RS1 while the fifth hub in the black system is called BS5. These designations did not change throughout data collection, thus RS3 in home H1 is the same physical piece of hardware as RS3 in home H5. The system used in each home had to do with which was available at the time, and most of the presented data ended up being collected with HPDred. Timestamp format is consistent across all data-types and is given in YY-MM-DD HH:MM:SS format with 24-hour time. Thus the file with name 2019-11-09_151604_RS1_H1.png represents an image from sensor hub 1(RS1) in H1, taken at 3:16:04 PM on November 9, 2019. Audio files are named based on the beginning second of the file, and so the file with name 2019-10-18_002910_BS5_H5.csv was captured from 12:29:10 AM to 12:29:19 AM on October 18, 2019 in H6 on hub 5 (BS5).

Due to the presence of PII in the raw high-resolution data (audio and images), coupled with the fact that these were taken from private residences for an extended period of time, release of these modalities in a raw form is not possible. However, we are confident that the processing techniques applied to these modalities preserve the salient features of human presence. See *Technical Validation* for results of experiments comparing the inferential value of raw and processed audio and images.

## Technical Validation

### Audio

In order to confirm that markers of human presence were still detectable in the processed audio data, we trained and tested audio classifiers on pre-labeled subsets of the collected audio data, starting with both unprocessed WAV files (referred to as P_0_ files) and CSV files that had gone through the processing steps described under *Data Processing* (referred to as P_1_ files).

Raw audio files were manually labeled as “noisy” if some sounds of human presence were audibly detectable (such as talking, movement, or cooking sounds) or “quiet”, if no sounds of human activity were heard. Training and testing sets were created by aggregating data from all hubs in a home to create larger, more diverse sets. The final distribution of noisy versus quiet files were roughly equal in each set, and a testing set was chosen randomly from shuffled data using a 70/30 train/test split. Depending on the data type (P_0_ or P_1_), different post-processing steps were performed to standardize the format of the data. Classification was done using a *k*-nearest neighbors (*k*-NN) algorithm. See Table 4 for classification performance on the two file types. The results show that while the predictive capabilities of the processed data are slightly lower than the raw counterpart, a simple model is still able to detect human presence most of the time. The exception to this is data collected in H6, which has markedly lower testing accuracy on the P_1_ data.

**Table 4:** Performance of a *k*-nearest neighbors classifier on unprocessed audio (P_0_), and audio data as publicly available in the database (P_1_).

Home	Sample Size		% Noisy	Accuracy	
	Train	Test		P_0_	P_1_
H1	2,682	1,142	0.59	0.89	0.99
H2	2,226	949	0.51	0.94	0.90
H3	5,255	2,245	0.38	0.91	0.90
H4	2,903	1,236	0.52	0.92	0.88
H5	3,026	1,289	0.61	0.93	0.88
H6	1,655	705	0.56	0.93	0.75

### Zone-level image ground truth

In order to make the downsized images most useful, we created zone based image labels, specifying if there was a human visible in the frame for each image in the released dataset. A pre-trained object detection algorithm, You Only Look Once - version 5 (YOLOv5)^26^, was used to classify the 112 × 112 pixel images as occupied or unoccupied. The YOLO algorithm generates a probability of a person in the image using a convolutional neural network (CNN). The optimal cut-off threshold that was used to classify an image as occupied or vacant was found through cross-validation and was unique for each hub. The median cut-off value was 0.3, though the values ranged from 0.2 to 0.6. Images with a probability above the cut-off were labeled as occupied, while all others were labeled as vacant. Images from both groups (occupied and vacant) were then randomly sampled, and the presence or absence of a person in the image was verified manually by the researchers. From these verified samples, we generated point estimates for: the probability of a truly occupied image being correctly identified (the sensitivity or true positive rate); the probability of a truly vacant image being correctly identified (the specificity or true negative rate); the probability of an image labeled as occupied being actually occupied (the positive predictive value or PPV); and the probability of an image labeled as vacant being actually vacant (the negative predictive value or NPV). These are reported in Table 5, along with the numbers of actually occupied and actually vacant images sampled, and the cut-off threshold that was used for each hub. The two sets of images (those labeled occupied and those labeled vacant by the YOLO algorithm) were each randomly sampled in an attempt to get an equal number of each type. Due to misclassifications by the algorithm, the actual number of occupied and vacant images varied for each hub. Since the subsets of labeled images were randomly sampled, a variety of lighting scenarios were present. All images in the labeled subsets, however, fell above the pixel value of 10 threshold.

**Table 5:** Accuracy metrics for the zone-based image labels.

Home	Hub	Thrsh	n Occ	n Vac	TPR	TNR	PPV	NPV
H1	RS1	0.3	99	101	0.95	0.94	0.94	0.95
	RS2	0.5	43	157	0.95	0.62	0.41	0.98
	RS3	0.3	98	102	0.98	0.96	0.96	0.98
	RS4	0.3	99	101	0.99	0.98	0.98	0.99
	RS5	0.3	94	106	1.00	0.94	0.94	1.00
	comb		433	567	0.98	0.86	0.85	0.98
H2	RS1	0.3	100	100	1.00	1.00	1.00	1.00
	RS2	0.3	103	97	0.97	1.00	1.00	0.97
	RS4	0.3	100	100	1.00	1.00	1.00	1.00
	RS5	0.3	108	92	0.93	1.00	1.00	0.92
	comb		411	389	0.97	1.00	1.00	0.97
H3	RS1	0.3	100	100	0.92	0.92	0.92	0.92
	RS2	0.2	77	123	0.99	0.80	0.76	0.99
	RS3	0.4	93	107	0.95	0.89	0.88	0.95
	RS4	0.5	91	109	0.96	0.88	0.87	0.96
	RS5	0.5	100	100	0.85	0.85	0.85	0.85
	comb		461	539	0.93	0.87	0.86	0.93
H4	RS1	0.3	102	98	0.98	1.00	1.00	0.98
	RS2	0.4	94	106	1.00	0.94	0.94	1.00
	RS3	0.35	99	101	0.95	0.94	0.94	0.95
	RS4	0.3	100	100	1.00	1.00	1.00	1.00
	RS5	0.3	97	103	1.00	0.97	0.97	1.00
	comb		492	508	0.99	0.97	0.97	0.99
H5	RS1	0.3	111	89	0.88	0.98	0.98	0.87
	RS2	0.5	86	114	0.92	0.82	0.79	0.93
	RS3	0.6	93	107	0.99	0.93	0.92	0.99
	RS4	0.3	100	100	1.00	1.00	1.00	1.00
	RS5	0.4	97	103	0.97	0.94	0.94	0.97
	comb		487	513	0.95	0.93	0.93	0.95
H6	BS2	0.3	94	106	1.00	0.94	0.94	1.00
	BS3	0.3	92	108	0.98	0.91	0.90	0.98
	BS4	0.5	101	99	0.94	0.95	0.95	0.94
	BS5	0.6	84	116	0.96	0.84	0.81	0.97
	comb		371	429	0.97	0.91	0.90	0.97

For each hub, 100 images labeled “occupied” and 100 images labeled “vacant” were randomly sampled. **Thrsh** gives the hub specific cut-off threshold that was used to classify the image as occupied or vacant, based on the output from the YOLOv5 algorithm. The number that were verified to be occupied and verified to be vacant are given in **n Occ** and **n Vac**. Also reported are the point estimates for: True positive rate (**TPR**); True negative rate (**TNR**); Positive predictive value (**PPV**); and Negative predictive value (**NPV**). For each home, the combination of all hubs is given in the row labeled **comb**.

**Fig. 7:**
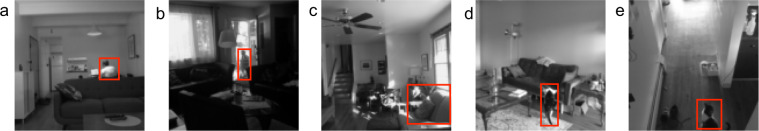
Five images that were misclassified by the YOLOv5 labeling algorithm. (**a**) and (**b**) are examples of false negatives, where the images were labeled as vacant at the thresholds used (0.3 and 0.4, respectively). (**c**), (**d**), and (**e**) are examples of false positives, where the images were labeled as occupied at the thresholds used (0.5, 0.3, and 0.6, respectively). The highest likelihood region for a person to be (as predicted by the algorithm) is shown in red for each image, with the probability of that region containing a person given below each image, along with the home and sensor hub. (**d**) and (**e**) both highlight cats as the most probable person location, which occurred infrequently.

Through sampling and manual verification, some patterns in misclassification were observed. For instance, false positives (the algorithm predicting a person was in the frame when there was no one) seemed to occur more often on cameras that had views of big windows, where the lighting conditions changed dramatically. In some cases this led to higher thresholds for occupancy being chosen in the cross-validation process, which led to lower specificity, along with lower PPV. An example of this is shown in Fig. 7c,where a vacant image was labeled by the algorithm as “occupied” at the cut-off threshold specified in Table 5. In other cases, false negatives were found to occur more often in cameras that had a long field of view, where people spent time far from the camera. Examples of these are given in Fig. 7a,b, which were labeled as vacant at the thresholds used.

**Fig. 8:**
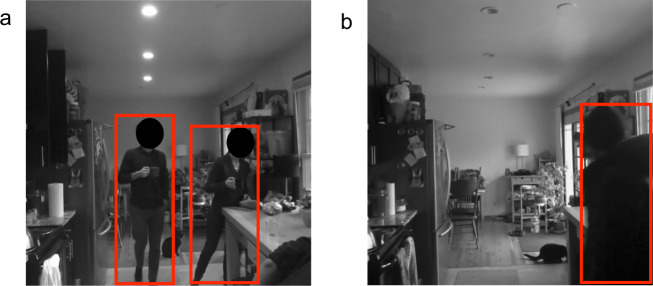
Overall the labeling algorithm had good performance when it came to distinguishing people from pets.

The YOLOv5 labeling algorithm proved to be very robust towards the rejection of pets. This is likely because the version of the algorithm used was pre-trained on the Common Objects in Context (or COCO) dataset^24^, which includes over 10,000 instances each of dogs and cats. Figure 8 gives two examples of correctly labeled images containing a cat. Some homes had higher instances of false positives involving pets (see Fig. 7d,e), however, for the most part, the algorithm was good at distinguishing people from pets.

**Fig. 9:**
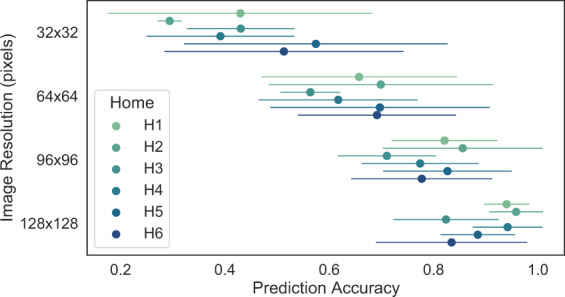
Effect of image resolution on prediction accuracy of the YOLOv5 algorithm. Points show the mean prediction accuracy of the algorithm on a roughly balanced set of labeled images from each home, while the error bars give the standard deviations of all observations for the home.

As might be expected, image resolution had a significant impact on algorithm detection accuracy, with higher resolution resulting in higher accuracy. To show the results of resolution on accuracy, we ran the YOLOv5 algorithm on balanced, labeled datasets at a variety of sizes (32 × 32 pixels up-to 128 × 128 pixels), and compared accuracy (defined as the total that were correctly identified divided by the total classified) across homes. The results are given in Fig. 9. To generate the different image sizes, the 112 × 112 images were either downsized using bilinear interpolation, or up-sized by padding with a white border, to generate the desired image size. Since higher resolution did have significantly better performance, the ground truth labeling was performed on the larger sizes (112 × 112), instead of the 32 × 32 sizes that are released in the database.

### Environmental sensors

**Fig. 10:**
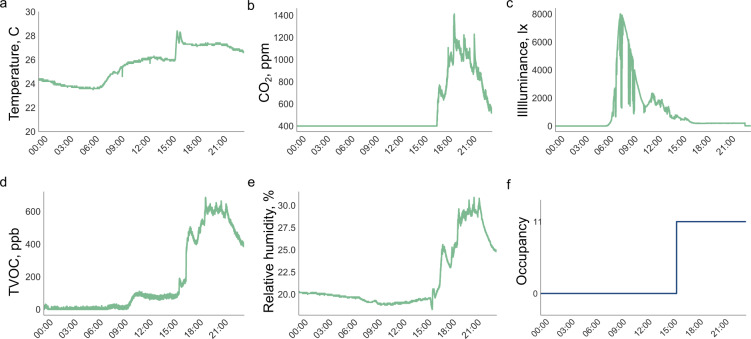
Time series environmental readings from one day (November 3, 2019) in H6, along with occupancy status.

The sensors used were chosen because of their ease of integration with the Raspberry Pi sensor hub. Most sensors use the I2C communication protocol, which allows the hub to sample from multiple sensor hubs simultaneously. All were inexpensive and available to the public at the time of system development. See Table 6 for sensor model specifics. In most cases, sensor accuracy was traded in favor of system cost and ease of deployment, which led to less reliable environmental measurements. Trends in the data, however, are still apparent, and changes in the state of a home can be easily detected by. See Fig. 10 for 24-hour samples of environmental data, along with occupancy. Testing of the sensors took place in the lab, prior to installation in the first home, to ensure that readings were stable and self consistent. However, formal calibration of the sensors was not performed.

**Table 6:** Hardware used in the data acquisition system.

Equipment	Make & Model	Use
Single-board computer (SBC)	Raspberry Pi 3	Data collection platform
Camera	Raspberry Pi Camera Module v2	Image collection
Microphone	Knowles SPH0645	Audio collection
Gas sensor	Sensirion SGP30	TVOC & eCO_2_ readings
Temperature and humidity sensor	Aosong DHT22	Temperature & rH readings
Light sensor	Avago APDS-9301	Illuminance readings
Battery pack	Romoss Solo 5 Power Bank	Supply short-term power to sensor hubs
Wireless router	Cradlepoint COR IBR600B	On-site network connection
Server	Antsle one Pro 8-Cores	On-site data storage

Reliability of the environmental data collection rate (system performance) was fairly good, with higher than 95% capture rate for most modalities. The temperature and humidity sensor had more dropped points than the other environmental modalities, and the capture rate for this sensor was around 90%. Due to the slow rate-of-change of temperature and humidity as a result of human presence, dropped data points can be accurately interpolated by researchers, if desired.

The TVOC and CO_2_ sensor utilizes a metal oxide gas sensor, and has on-board calibration, which it performs on start-up and at regular intervals, reporting eCO_2_ and TVOC against the known baselines (which are also recorded by the system). The temperature and humidity sensor is a digital sensor that is built on a capacitive humidity sensor and thermistor. The sensor is calibrated prior to shipment, and the readings are reported by the sensor with respect to the calibration coefficient that is stored in on-board memory. The illuminance sensor uses a broadband photodiode and infrared photodiode, and performs on-board conversion of the analog signal to a digital signal, meant to approximate the human eye response to the light level.

Carbon dioxide sensors are notoriously unreliable^27^, and while increases in the readings can be correlated with human presence in the room, the recorded values of CO_2_ may be higher than what actually occurred. Spatial overlap in coverage (i.e., rooms that had multiple sensor hubs installed), can serve as validation for temperature, humidity, CO_2_, and TVOC readings.

In addition to the environmental sensors mentioned, a distance sensor that uses time-of-flight technology was also included in the sensor hub. The sensor was supposed to report distance of the nearest object up to 4 m. The actual range it can report, however, is subject to an internal mode selection and is heavily impacted by ambient light levels. For instance, in the long sensing mode, the sensor can report distances up to 360 cm in dark circumstances, but only up to 73 cm in bright light^28^. Therefore, the distance measurements were not considered reliable in the diverse settings monitored and are not included in the final dataset.

### Ground truth

Verification of the ground truth was performed by using the image detection algorithms developed by the team. After training highly accurate image classifiers for use in the ARPA-E SENSOR project, these algorithms were applied to the full collected image sets to generate binary decisions on each image, declaring if the frame was occupied or vacant. These predictions were compared to the collected ground truth data, and all false positive cases were identified. False positive cases, (i.e., when the classifier thinks someone is in the image but the ground truth says the home is vacant) may represent a mislabeled point. The images from these times were flagged and inspected by a researcher. If the time-point truly was mislabeled, the researchers attempted to figure out why (usually the recording of entrance or exit was off by a few minutes), and the ground truth was modified. The fact that all homes had cameras facing the main entrance of the home made it simple to correct these cases after they were identified. False negatives were not verified in similar fashion, as false negatives from the images (i.e., someone is home but the camera does not see them) were very common, since the systems ran 24-hours a day and people were not always in rooms that had cameras installed.

### System reliability

Each home was to be tested for a consecutive four-week period. Data collection was checked roughly daily, either through on-site visits or remotely. Due to technical challenges encountered, a few of the homes’ testing periods were extended to allow for more uninterrupted data acquisition. Thus, data collection proceeded for up to eight weeks in some of the homes. Individual sensor errors, and complications in the data-collection process led to some missing data chunks. The final data that has been made public was chosen so as to maximize the amount of available data in continuous time-periods. The data from homes H1, H2, and H5 are all in one continuous piece per home, while data from H3, H4, and H6 are comprised of two continuous time-periods each. For a number of reasons, the audio sensor has the lowest capture rate. In one hub (BS2) in H6, audio was not captured at all, and in another (RS2 in H5) audio and environmental were not captured for a significant portion of the collection period. Overall, audio had a collection rate of 87%, and environmental readings a rate of 89% for the time periods released. If not considering the two hubs with missing modalities as described, the collection rates for both of these are above 90%. Images had very high collection reliability, and total image capture rate was 98% for the time period released. Dark images (not included in the dataset), account for 19–40% of images captured, depending on the home. See Table 3 for a summary of the collection reliability, as broken down by modality, hub, and home.

## Usage Notes

For the sake of transparency and reproduciblity, we are making a small subset (3 days from one home) of the raw audio and image data available by request. Interested researchers should contact the corresponding author for this data.

## Data Availability

All code used to collect, process, and validate the data was written in Python and is available for download^29^ (https://github.com/mhsjacoby/HPDmobile). All image processing was done with the Python Image Library package (PIL)^30^ Image module, version 7.2.0. Audio processing was done with SciPy^31^ io module, version 1.5.0. Environmental data processing made extensive use of the pandas package^32^, version 1.0.5. The code base that was developed for data collection with the HPDmobile system utilizes a standard client-server model, whereby the sensor hub is the server and the VM is the client. Note that the term “server” in this context refers to the SBC (sensor hub), and not the the on-site server mentioned above, which runs the VMs. All collection code on both the client- and server-side were written in Python to run on Linux systems. Technical validation of the audio and images were done in Python with scikit-learn^33^ version 0.24.1, and YOLOv5^26^ version 3.0.
